# Beta 3 Adrenergic Receptor Activation Rescues Metabolic Dysfunction in Female Estrogen Receptor Alpha-Null Mice

**DOI:** 10.3389/fphys.2019.00009

**Published:** 2019-02-05

**Authors:** Stephanie L. Clookey, Rebecca J. Welly, Dusti Shay, Makenzie L. Woodford, Kevin L. Fritsche, R. Scott Rector, Jaume Padilla, Dennis B. Lubahn, Victoria J. Vieira-Potter

**Affiliations:** ^1^Department of Nutrition and Exercise Physiology, University of Missouri, Columbia, MO, United States; ^2^Harry S. Truman Memorial Veterans’ Hospital, Columbia, MO, United States; ^3^Department of Medicine, University of Missouri, Columbia, MO, United States; ^4^Dalton Cardiovascular Research Center, University of Missouri, Columbia, MO, United States; ^5^Child Health, University of Missouri, Columbia, MO, United States; ^6^Department of Biochemistry, University of Missouri, Columbia, MO, United States

**Keywords:** adipose tissue, energy expenditure, browning, insulin resistance, high fat diet, CL 316, 243, obesity, rodent

## Abstract

Metabolic disease risk escalates following menopause. The mechanism is not fully known, but likely involves reduced signaling through estrogen receptor alpha (ERα), which is highly expressed in brown and white adipose tissue (BAT and WAT).

**Objective:** Test the hypothesis that uncoupling protein (UCP1) activation mitigates metabolic dysfunction caused by loss of signaling through ERα.

**Methods:** At 8 weeks of age, female ERα knock out (KO) and wild-type mice were housed at 28°C and fed a Western-style high-fat, high sucrose diet (HFD) or a normal low-fat chow diet (NC) for 10 weeks. During the final 2 weeks, they received daily injections of CL 316,256 (CL), a selective β3 adrenergic agonist, or vehicle control (CTRL), creating eight groups: WT-CTRL, WT-CL, KO-CTRL, and KO-CL on HFD or NC; *n* = 4–10/group.

**Results:** ERαKO demonstrated exacerbated HFD-induced adiposity gain (*P* < 0.001) and insulin resistance (*P* = 0.006). CL treatment improved insulin sensitivity (*P* < 0.05) and normalized ERαKO-induced adiposity increase (*P* < 0.05). In both genotypes, CL increased resting energy expenditure (*P* < 0.05) and induced WAT beiging indicated by increased UCP1 protein in both perigonadal (PGAT) and subcutaneous (SQAT) depots. These effects were attenuated under HFD conditions (*P* < 0.05). In KO, CL reduced HFD energy consumption compared to CTRL (*P* < 0.05). Remarkably, CL increased WAT ERβ protein levels of both WT and KO (*P* < 0.001), revealing CL-mediated changes in estrogen signaling may have protective metabolic effects.

**Conclusion:** CL completely restored metabolic dysfunction in ERαKO mice. Thus, UCP1 may be a therapeutic target for treating metabolic dysfunction following loss of estrogen receptor signaling.

## Introduction

Compared to males, ovary-intact females are protected against obesity and its associated metabolic consequences, including insulin resistance, which precedes diabetes onset (Brand et al., 2012). However, postmenopausal women are more likely to be obese (Auro et al., 2014; Stefanska et al., 2015) and are twice as likely to develop type 2 diabetes compared to men and younger women (Klöting et al., 2010). Reducing metabolic disease risk in this vulnerable population is an urgent public health priority (Tchernof et al., 1998; Spritzer and Oppermann, 2013; Stefanska et al., 2015). While ovarian hormones (notably, estrogen) are known to be metabolically protective (Klöting et al., 2010), the mechanism(s) remain largely unknown.

Adipose tissue is heavily influenced by estrogen (Ogden et al., 2012; Davis et al., 2013) and is an important target tissue to improve metabolic health. Importantly, it is a vital glucose-regulator (Lomonaco et al., 2012) especially among women who generally have greater relative fat mass (Wade et al., 1985). In fact, insulin-mediated adipose tissue glucose uptake predicted systemic insulin sensitivity following ovariectomy (OVX) in a rodent model used to mimic the effects of menopause (Park Y.M. et al., 2015). Pinpointing what factors protect female adipose tissue prior to menopause could have tremendous implications for women’s health. In this regard, the protection against adipose tissue dysfunction is likely due to adipocyte-specific estrogen signaling (D’Eon et al., 2005; Mauvais-Jarvis et al., 2013; Kim et al., 2014; Luglio, 2014) through estrogen receptor alpha (ERα) (Davis et al., 2013). Signaling through this steroid receptor has been shown to increase mitochondrial function and biogenesis in adipose tissue (Klinge, 2008), consistent with a signature profile of healthy adipose tissue ‘immunometabolism’ (Zidon et al., 2018). On the other hand, directly decreasing adipocyte ERα signaling causes adipocyte dysfunction (Davis et al., 2013).

A specific kind of adipose tissue typified by its high mitochondrial content and activity, the brown adipose tissue (BAT), and the more recently appreciated “beige” adipose tissue, play important roles in improving whole body metabolic function (Bartelt and Heeren, 2014). Notably, ovary-intact females, who are more insulin sensitive and protected against metabolic dysfunction, also have more BAT relative to total adiposity (Cypess et al., 2009; Pfannenberg et al., 2010; Ouellet et al., 2011; Wang et al., 2011) and may be more responsive than males to white adipose tissue “beiging” (Himms-Hagen et al., 1994; Kim et al., 2016), a process by which WAT increases its mitochondrial content and expression of key signature BAT proteins [e.g., uncoupling protein 1 (UCP1)]. Thus, estrogen may mediate WAT beiging.

Via ERα signaling, estrogen has been shown to be protective against high fat diet (HFD)-induced insulin resistance (Riant et al., 2009), suggesting that the ERα pathway may be a viable strategy to mitigate diet-induced metabolic dysfunction. ERα is also protective against HFD-induced metabolic inflammation (Morselli et al., 2014), whereas HFD suppresses ERα in adipose tissues (Gorres et al., 2011) and the central nervous system (Scudiero and Verderame, 2017). Further, suppression of hepatic ERα in mice exacerbates their response to HFD-induced hepatic insulin resistance (Zhu et al., 2014).

The chemical ligand, CL 316,243 (Bloom et al., 1992) (i.e., CL) is a highly selective systemic β_3_ adrenergic receptor agonist known to induce beiging of WAT. CL induces ectopic expression of UCP1 in WAT and improves insulin sensitivity (Borst and Hennessy, 2001; Poher et al., 2015). The mechanism by which CL reduces insulin resistance has not been fully elucidated. CL has also been shown to stimulate insulin secretion (Pang et al., 2010), which may contribute to its ability to increase glucose uptake; however, CL has been shown to reduce obesity-associated hyperinsulinemia (Ghorbani and Himms-Hagen, 1997). We and others have demonstrated that lack of UCP1 reduces insulin sensitivity (Winn et al., 2017). Thus, the insulin sensitizing effect of CL may involve its ability to increase UCP1. However, UCP1 is not required for all of the metabolic benefits of CL (Granneman et al., 2003). Whether or not CL’s metabolic benefits require ERα is not known. To test this hypothesis, we determined the effectiveness of CL to reduce obesity and insulin resistance in the presence and absence of ERα expression. We used ERαKO mice because ERα is diminished following menopause in women (Park et al., 2017), and the phenotype of metabolic dysfunction in ERαKO mice mimics that of postmenopausal women and OVX rodents. We compared mice null (i.e., whole body knock-out [KO]) for ERα expression (ERαKO) to littermate wild-type controls for their responsiveness to HFD-induced metabolic dysfunction, and the possible protective role of CL. We confirm that young otherwise healthy ERαKO mice have an increased susceptibility to HFD-induced obesity compared to WT littermate controls (Heine et al., 2000; Ohlsson et al., 2000 #122) and further demonstrate that CL mitigates metabolic dysfunction caused by loss of ERα and HFD-induced obesity.

## Materials and Methods

### Ethics Statement

All animal husbandry and experimental procedures were carried out in accordance with the AAALAC International and approved by the University of Missouri Institutional Animal Care and Use Committee.

### Animals and Experimental Design

**FIGURE 1:**
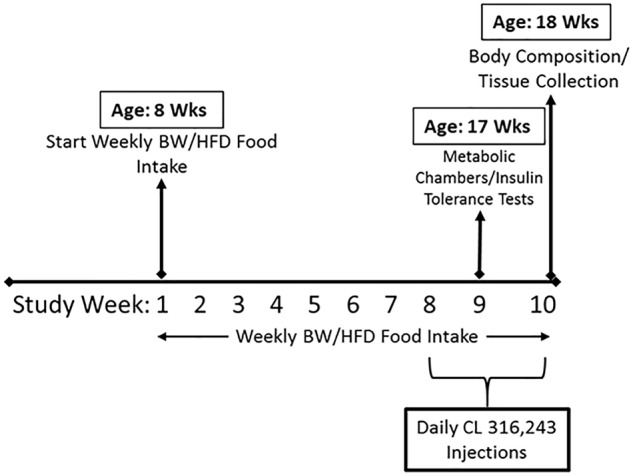
Schematic of study timeline. ERαKO and WT female mice were fed a normal chow diet or a high-fat diet for 10 weeks, then treated with selective β3 agonist, CL 316,243, for 2 weeks. Assessments were made for differences in food intake, weekly body weight, energy expenditure, insulin tolerance, and body composition prior to sacrifice. Posthumously, tissues were harvested and analyzed.

Heterozygote ERα-/+ mice on a C57BL/6J background were bred at our facility to produce homozygote (ERα-/-) and littermate wild-type mice, as previously described (Lubahn et al., 1993; Eddy et al., 1996). Briefly, development of the ERα-/- (i.e., ERαKO) mouse was accomplished by homologous recombination and insertion of a neomycin sequence containing premature stop codons and polyadenylation sequences into a Not one site in exon 2 of the mouse estrogen receptor gene. At 8 weeks of age, mice were fed a high sucrose, HFD consisting of 46.4% kcal from fat, 36% carbohydrate, and 17.6% protein, with a density of 4.68 kcal per gram (Test Diet, St. Louis, MO, United States, #1814692), or a normal chow (NC) diet (3.3 kcal/g of food, 13% kcal fat, 57% kcal carbohydrate, and 30% kcal protein, LabDiet, St. Louis, MO, United States, #5001) for a total of 10 weeks. Animals were housed two to three per cage (within group) at 28°C (i.e., thermoneutral conditions), in a light cycle from 0700 to 1900. At 16 weeks of age, animals began a regiment of daily intraperitoneal injections of CL 316,243 (#C5976, Sigma-Aldrich) or equal dose of saline control at 7 am during the final 2 weeks of the study. CL 316,243 compound (CL) was put in solution using deionized water, and was administered at a dose of 1 μg/g body weight. Thus, the study consisted of eight groups (*n* = 4–5/group for NC-fed animals, 10/group for HFD-fed animals): WT/control (CTRL), WT/CL, ERαKO/CTRL, ERαKO/CL either on HFD or NC. After a total of 10 weeks on each respective diet, with CL administration during the last 2 weeks, the animals were euthanized at 18 weeks of age following a 5-h fast. Blood and tissues were collected. Tissues were harvested and either fixed in 10% formalin or snap-frozen in liquid nitrogen and stored at -80°C until analyses. The basic study design including timeline is depicted in Figure 1.

### Body Composition and Tissue Weights

Body fat percentage (BF%) was measured by a nuclear magnetic resonance imaging whole-body composition analyzer (EchoMRI 4 in 1/1100; Echo Medical Systems, Houston, TX), 1 week prior to sacrifice. Upon sacrifice, interscapular BAT, subcutaneous (inguinal) WAT (SQAT), and visceral (perigonadal) WAT (PGAT) were extracted and tissue weights were collected.

### Energy Expenditure Assessments

Indirect calorimetry was utilized when mice were 17 weeks of age, after 1 week of CL treatment. HFD-fed animals (*n* = 10/group) were placed in indirect calorimetry chambers (Promethion; Sable Systems International, Las Vegas, Nevada) to assess metabolic activity parameters including total energy expenditure (TEE), resting energy expenditure (REE), respiratory quotient estimation (RQ), and spontaneous physical activity (SPA) by the summation of *x*-, *y*-, and *z-axis* beam breaks. Body weight and food intake were measured before and after each 48-h assessment. Each 48-h run captured at least two light and two dark cycles of REE.

### Insulin Tolerance Testing (ITT)

Insulin tolerance tests were performed on HFD-fed animals at 17 weeks of age, 5 days before sacrifice (*n* = 10/group). After a 5-h fast, blood glucose was measured from the tail vein (i.e., time 0), and blood was sampled by a glucometer (Alpha Trak, Abbott Labs). Insulin was administered at a dose of (0.7 U/kg body weight (BW)) via intraperitoneal injection, as previously described (Vieira Potter et al., 2012). Glucose measures were taken 30, 45, 60 and 120 min after the insulin injections, however, an abbreviated ITT curve is presented due to incidences of hypoglycemia during testing despite the reduced insulin dose. Glucose area under curve (AUC) from baseline was calculated.

### Fasting Blood Parameters

Plasma insulin, glucose, and non-esterified fatty acids (NEFA) assays were performed by a commercial laboratory (Comparative Clinical Pathology Services, Columbia, MO, United States) on an Olympus AU680 automated chemistry analyzer (Beckman-Coulter, Brea, CA, United States) using assays as per manufacturer’s guidelines. The homeostasis model assessment of insulin resistance (HOMA-IR) was used as a surrogate measure of systemic insulin resistance [(fasting insulin (μU/l) x fasting glucose (mg/dl)/405.1) (Matthews et al., 1985)] and indices of adipose tissue insulin resistance (ADIPO-IR) were calculated as the product of fasting insulin (μU/l) and fasting NEFAs (mmol/l) (Lomonaco et al., 2012). Fasting levels of circulating adiponectin were measured using colorimetric ELISA (Quantikine MRP300; R&D Systems, Minneapolis, MN, United States) and data are presented as ng/ml.

### Histological Assessments

Formalin-fixed samples were processed through paraffin embedment, sectioned at 5 μm (interscapular BAT, visceral (PGAT) and subcutaneous (SQAT) WAT) and stained in a 1:1200 dilution with UCP1 antibody (#U6382, 1:1000, Sigma-Aldrich; secondary antibody, #K400311-2, Envision Rabbit, Agilent) for 30 min with a heat-induced epitope retrieval (HIER) pretreatment, using DAKO brand citrate in a decloaking chamber. Sections were evaluated via an Olympus BX34 photomicroscope (Olympus, Melville, NY) and images were taken via an Olympus SC30 Optical Microscope Accessory CMOS color camera. Adipocyte size was calculated from three independent regions of the same 40× objective fields for SQAT, PGAT, and interscapular BAT depots (50 adipocytes/animal). Cross-sectional areas of the adipocytes were obtained from perimeter tracings using Image J software as previously described (Wainright et al., 2015). An investigator blinded to the groups performed all procedures.

### RNA Extraction and Quantitative Real-Time RT-PCR

Interscapular BAT samples were homogenized in TRIzol solution using a tissue homogenizer (TissueLyser LT, Qiagen, Valencia, CA, United States). Total RNA was isolated according to the Qiagen’s RNeasy lipid tissue protocol and assayed using a Nanodrop spectrophotometer (Thermo Scientific, Wilmington, DE, United States) to assess purity and concentration. First-strand cDNA was synthesized from total RNA using the High Capacity cDNA Reverse Transcription kit (Applied Biosystems, Carlsbad, CA, United States). Quantitative real-time PCR was performed as previously described using the ABI Step One Plus sequence detection system (Applied Biosystems) (Roseguini et al., 2010; Padilla et al., 2013). All primers were purchased from IDT (Coralville, IA, United States) and Sigma Aldrich (St. Louis, MO, United States). Housekeeping gene cycle threshold (CT) was not different among the groups of animals. mRNA expression was calculated by 2^ΔCT^ where ΔCT = Housekeeping gene CT – gene of interest CT and presented as fold-difference. mRNA levels were normalized to the WT/CTRL group, which was set at 1. All primer sequences are provided in Table 1.

**Table 1:** Primer sequences.

Primer	Forward	Reverse	Company
UCP1	CACGGGGACCTACAATGCTT	ACAGTAAATGGCAGGGGACG	IDT
CD11c	ATGCCACTGTCTGCCTTCAT	GAGCCAGGTCAAAGGTGACA	IDT
TNFa	CTATGTCTCAGCCTCTTCTC	CATTTGGGAAACTTCTCATCC	Sigma
ER alpha	CAAGGTAAATGTGTGGAAGG	GTGTACACTCCGGAATTAAG	Sigma
ER beta	CTCAACTCCAGTATGTACCC	CATGAGAAAGAAGCATCAGG	Sigma
B actin	GATGTATGAAGGCTTTGGTC	TGTGCACTTTTATTGGTCTC	Sigma
18 s	TCAAGAACGAAAGTCGGAGG	GGACATCTAAGGGCATCAC	IDT
RPS13	TGCCGTTTCCTACCTCGTTC	CACGTCGTCAGACGTCAACT	IDT

### Western Blotting

Protein was measured as previously described (Winn et al., 2017). Briefly, protein samples (10 μg/lane) were separated by SDS–PAGE, transferred to polyvinylidene difluoride membranes, and probed with primary antibodies, including UCP1 (#U6382, 1:1000; Sigma-Aldrich), UCP2 (#89326, 1:1000; Cell Signaling), ERα (#75635, 1:1000, Abcam), and ERβ (#AB3577, 1:2000, Abcam). Intensity of individual protein bands were quantified using FluoroChem HD2 (AlphaView, version 3.4.0.0), and expressed relative to the housekeeping protein, beta-tubulin. Amido Black was used as an additional control of total protein loading.

### Statistics

A 2×2×2 analysis of variance (ANOVA) was used to evaluate the effects of genotype (G, ERαKO vs. WT), treatment (T, CL vs. CTRL), and diet (D, NC vs. HFD), as well as treatment interactions. Where appropriate, Tukey’s *post hoc* tests were used to indicate significant between-group differences with the following symbols: ^∗^ different from all other groups, # different from all other groups except NC WT/CL, $ different from all other groups except HFD KO/CL, 2 different from NC WT/CL, 4 different from NC KO/CL, 5 different from HFD WT/CTRL, 6 different from HFD WT/CL, 7 different from HFD KO/CTRL, 8 different from HFD KO/CL. All data are presented as mean ± standard error of the mean (SEM). For all statistical tests, significance was accepted at *P* < 0.05. All statistical analyses were performed with SPSS V25.0.

## Results

### ERα Ablation Exacerbates HFD Induced Metabolic Dysfunction

**FIGURE 2:**
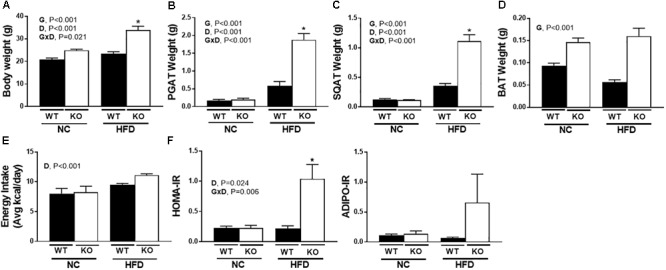
ERα ablation exacerbates high-fat diet induced metabolic dysfunction. ERαKO and WT female mice were fed a normal chow diet or a high-fat diet. Assessments were made for differences in **(A)** body weight, **(B)** perigonadal adipose tissue (PGAT) weight, **(C)** subcutaneous adipose tissue (SQAT) weight, **(D)** brown adipose tissue (BAT) weight, **(E)** energy intake, **(F)** HOMA-IR, **(G)** ADIPO-IR. WT, wild-type; KO, ERα knock-out; NC, normal chow; HFD, high fat diet; data are expressed as means ± standard error (SE); *n* = 4–10/group. *G* = significant main effect of genotype, *P* < 0.05. *D* = significant main effect of diet, *P* < 0.05. G×D = significant interaction between genotype and diet, *P* < 0.05. ^∗^*P* < 0.05 compared to all other groups.

Consistent with many previous studies (Ohlsson et al., 2000; Ribas et al., 2010; Gao and Dahlman-Wright, 2013), female ERαKO were heavier than WT (G, *P* < 0.001) and both genotypes increased body weight under conditions of HFD feeding (D, *P* < 0.001). However, ERαKOs experienced a greater increase in body weight in response to HFD than WT (G×D, *P* = 0.002) (Figure 2A). Increases in adiposity coincided with differences in body weight such that ERαKOs had significantly greater perigonadal (i.e., PGAT, a visceral depot) adipose tissue and subcutaneous inguinal adipose tissue (SQAT) following consumption of the HFD when compared to WT mice (G×D, *P* < 0.001) (Figure 2B,C). BAT depot weight in the ERαKO was greater than that in the WT mice, possibly due to increased lipid accumulation or ‘BAT whitening’ (G, *P* < 0.001) (Figure 2D) compared to BAT of WT mice both on either diet. Although they had increased body weight and adiposity, the ERαKOs did not consume more calories than WT when fed a NC diet; however, both genotypes increased caloric consumption under HFD conditions, as expected (D, *P* < 0.001) (Figure 2E). Furthermore, unlike WT, ERαKOs exhibited evidence of insulin resistance via elevated HOMA-IR in response to HFD (G×D, *P* = 0.006) (Figure 2F). The same trend was observed in ADIPO-IR, though not significant (Figure 2G). The increase in those surrogate markers of insulin resistance were primarily driven by hyperinsulinemia in the ERaKOs in response to HFD; ERaKOs fed HFD exhibited a fourfold increase in fasting insulin compared to NC (G×D, *P* = 0.036) (Table 2). This coincided with HFD-associated reductions in non-esterified fatty acids (NEFAs) in circulation (D, *P* = 0.03) likely due to insulin-mediated suppression of lipolysis. Similarly, fasting glucose was significantly elevated in ERαKO (but not WT) under HFD conditions (G×D, *P* = 0.009) (Table 2). Thus, ERα ablation increases susceptibility to HFD-induced metabolic dysfunction.

**Table 2:** Blood biochemistry.

Diet	Group	Adiponectin (ng/mL)	Insulin (ng/ml)	Glucose (mg/dl)	NEFA (mmol/L)
NC	WT Ctrl	12628.50 ± 1691.48	0.46 ± 0.09	211.25 ± 22.02	0.26 ± 0.06
	WT CL	11807.75 ± 446.48	0.79 ± 0.30	241.25 ± 16.12	0.13 ± 0.03
	KO Ctrl	7419 ± 857.18	0.51 ± 0.06	180.75 ± 35.32	0.26 ± 0.07
	KO CL	7809.25 ± 2147.32	0.46 ± 0.05	115.00 ± 24.09	0.13 ± 0.04
HFD	WT Ctrl	9903.60 ± 726.41	0.46 ± 0.07	179.90 ± 16.21	0.14 ± 0.04
	WT CL	12819.18 ± 1011.91	0.81 ± 0.13	178.27 ± 16.16	0.09 ± 0.02
	KO Ctrl	9513.50 ± 841.55	2.05 ± 0.58	222.00 ± 25.10	0.17 ± 0.06
	KO CL	13566.44 ± 1058.08	1.11 ± 0.14	180.60 ± 24.14	0.06 ± 0.01
*P*-value		G, *P* = 0.013; G×D, *P* = 0.008; T×D, *P* = 0.037	D, *P* = 0.031; G×D, *P* = 0.036	G×D, *P* = 0.009	T, *P* = 0.004; D, *P* = 0.03

*G, *P* < 0.05 main effect of genotype; T, *P* < 0.05 main effect of CL treatment; D, *P* < 0.05 main effect of diet; G×T *P* < 0.05 G, T interaction; G×D *P* < 0.05 G, D interaction; T×D *P* < 0.05 T, D interaction; G×T×D *P* < 0.05 G, T, D interaction.*

### CL Rescues HFD-Induced Metabolic Dysfunction in the ERαKO

**FIGURE 3:**
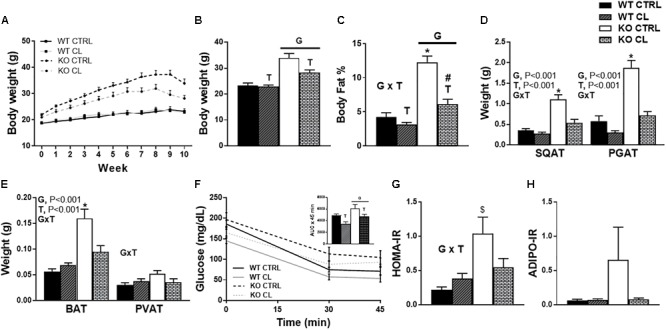
CL 316,243 rescues high-fat diet induced metabolic dysfunction in ERαKO. ERαKO and WT female mice were fed a normal chow diet or a high-fat diet for 10 weeks, then treated with selective β3 agonist, CL 316,243, for 2 weeks. Assessments were made for differences in **(A)** change in body weight, **(B)** final body weight, **(C)** body fat percentage, **(D)** white adipose tissue depot weights, **(E)** brown adipose tissue depot weights, **(F)** insulin tolerance test performance, **(G)** HOMA-IR, **(H)** ADIPO-IR. WT, wild-type; KO, ERα knock-out; CTRL, saline control; CL, CL 316,243 treatment; data are expressed as means ± standard error (SE); *n* = 10/group. *G* = significant main effect of genotype, *P* < 0.05. *T* = significant main effect of CL, *P* < 0.05, G×T = significant interaction between genotype and treatment, *P* < 0.05. ^∗^ *P* < 0.05 compared to all other groups; ^#^ compared to all except NC WT/CL; ^$^ compared to all except HFD KO/CL.

As discussed above, ERαKO animals exhibited greater increases in body weight compared to WT under both dietary conditions (G, *P* < 0.001); however, the CL treatment mitigated their HFD-induced weight gain (T, *P* = 0.009) (Figure 3A,B). Whereas ERα ablation increased adiposity (G, *P* < 0.001), CL induced a significant adiposity reduction (T, *P* < 0.001) (Figure 3C). While ERαKO animals gained more weight on HFD, they were also more responsive to CL-induced decreases in adiposity than WTs (G×T, *P* < 0.05) (Figure 3C). Likewise, ERαKO had greater CL-induced reductions in SQAT and PGAT depot weights relative to WT (G×T, *P* < 0.001 for both) (Figure 3D). ERα ablation also increased BAT weight in both interscapular and periaortic fat depots, possibly indicative of a “whitening” of BAT; however, this increase was normalized with CL (G×T, *P* < 0.001) (Figure 3E).

ERαKO animals had impaired insulin tolerance compared to WT littermates (G, *P* = 0.011), whereas CL treatment decreased glucose AUC during ITT (T, *P* = 0.018) (Figure 3F), normalized HOMA-IR in the KO to those of WT CTRL (G×T, *P* < 0.05) (Figure 3G), and tended to improve ADIPO-IR (Figure 3H). However, it is important to note that CL treatment caused hypoglycemia in a subset of animals (WT *n* = 8; KO *n* = 5) which prevented full ITT curves from being determined in those animals. No animals in the CTRL groups experienced hypoglycemia. Although this confirms the robust insulin-sensitizing effect of CL, future studies should perform more precise measures of insulin sensitivity in order to validate our suggested findings. The CL-induced hypoglycemia during the ITT may have been attributed to the effect of CL to increase insulin secretion, but since insulin levels were not measured throughout the ITT, this is an important assessment that should be done in future studies. Thus, CL reduced weight gain and adiposity, and improved insulin sensitivity in both ERαKO and WT animals on HFD, effectively normalizing metabolic health.

### CL Increases Energy Expenditure and Attenuates Energy Consumption in HFD-fed ERαKO Mice

**FIGURE 4:**
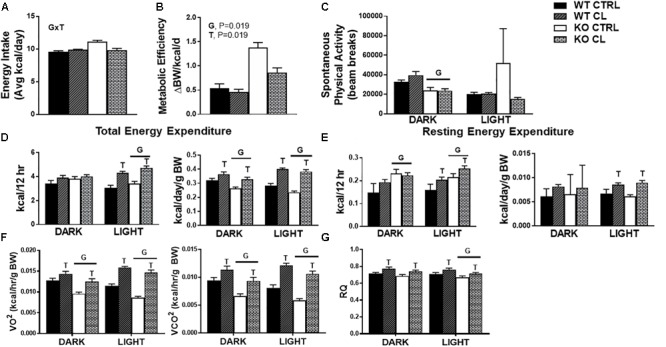
CL 316,243 increases energy expenditure and attenuates energy consumption in HFD-fed ERαKO. ERαKO and WT female mice were fed a normal chow diet or a high-fat diet for 10 weeks, then treated with selective β3 agonist, CL 316,243, for 2 weeks. Assessments were made for differences in **(A)** energy intake, **(B)** metabolic efficiency, **(C)** spontaneous physical activity, **(D)** total energy expenditure, **(E)** resting energy expenditure, **(F)** VO^2^ and VCO^2^ consumption, **(G)** RQ values. WT, wild-type; KO, ERα knock-out; CTRL, saline control; CL, CL 316,243 treatment; data are expressed as means ± standard error (SE); *n* = 10/group. *G* = significant main effect of genotype, *P* < 0.05. *T* = significant main effect of CL, *P* < 0.05. G×T = significant interaction between genotype and treatment, *P* < 0.05.

There was no influence of CL on energy intake in WT mice under either dietary condition (Supplementary Figure S1), yet CL attenuated HFD energy overconsumption in the ERαKO animals (G×T, *P* = 0.04) (Figure 4A). CL also decreased metabolic efficiency in the ERαKOs (G×T, *P* = 0.019) (Figure 4B), yet did not affect cage (i.e., spontaneous) physical activity (SPA) (Figure 4C). Similarly, CL increased total energy expenditure (TEE) (T, *P* < 0.05) (Figure 4D) by increasing resting energy expenditure (REE) (T, *P* < 0.05) (Figure 4E). The EE data were generated based on VO^2^ and VCO^2^ (Figure 4F) which indicate that KO reduced and CL increased these measures. CL treatment also increased RQ (Figure 4G), suggestive of a shift away from oxidative and toward glycolytic metabolism.

### CL Induced WAT Beiging Differs in WT and ERαKO Mice

**FIGURE 5:**
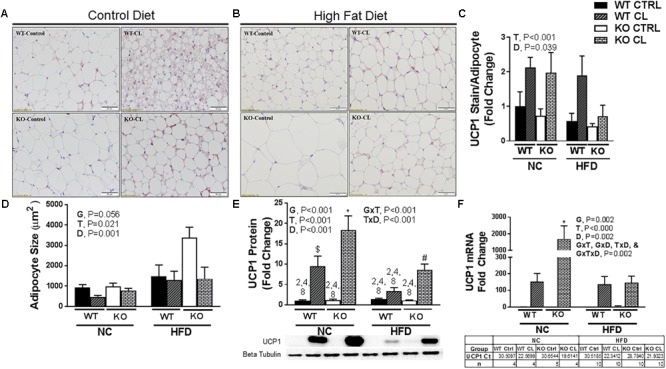
CL 316,243induces SQAT browning in WT and ERαKO mice. High-fat diet fed ERαKO and WT female mice were treated with selective β3 agonist, CL 316,243, for 2 weeks. Assessments were made for differences in **(A)** NC, UCP1-positive stained subcutaneous adipose tissue (SQAT) histology, **(B)** HFD, UCP1-positive stained SQAT, **(C)** UCP1 stain per adipocyte, **(D)** adipocyte size (μm), **(E)** UCP1 protein expression, **(F)** UCP1 mRNA expression. WT, wild-type; KO, ERα knock-out; CTRL, saline control; CL, CL 316,243 treatment; NC, normal chow; HFD, high fat diet; data are expressed as means ± standard error (SE); *n* = 4–10/group. *G* = significant main effect of genotype, *P* < 0.05. *T* = significant main effect of CL, *P* < 0.05. *D* = significant main effect of diet, *P* < 0.05. G×T = significant interaction between genotype and treatment, *P* < 0.05. G×D = significant interaction between genotype and diet, *P* < 0.05. G×T×D = significant interaction between genotype, treatment, and diet, *P* < 0.05. ^∗^ *P* < 0.05 compared to all other groups; ^#^ compared to all except NC WT/CL; ^$^ compared to all except HFD KO/CL.

Under NC dietary conditions, assessments of UCP1-stained SQAT [i.e., the WAT depot thought to be most susceptible to beiging (Himms-Hagen et al., 1994)] revealed a CL-induced multi-locular phenotype in both WTs and KOs, which appeared more robust in the lean WT compared to the more insulin resistant ERαKO (Figure 5A). Under HFD conditions, histological analysis did not show differences in CL-induced UCP1+ staining between WT and KO (Figure 5B). Overall, there was a main effect of CL treatment (*P* < 0.001) (Figure 5C), and HFD increased mean adipocyte size (*P* = 0.001) (Figure 5D) and attenuated CL-induced beiging (T×D, *P* < 0.001) (Figure 5E). Interestingly, assessments of UCP1 protein (Figure 5E) and mRNA (Figure 5F) indicated that the ERαKO were more responsive to CL (G×T, *P* < 0.01 for both protein and mRNA) under NC conditions. An additional finding was that HFD reduced basal (i.e., untreated) levels of SQAT UCP1 staining (*P* = 0.039, Figure 5C), protein (*P* < 0.001, Figure 5E) and mRNA (*P* = 0.002, Figure 5F) in both KO and WT mice.

**FIGURE 6:**
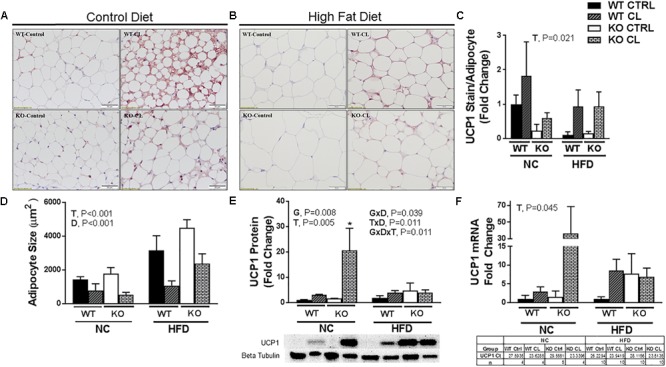
High-fat diet impairs CL 316,243-induced PGAT browning. High-fat diet fed ERαKO and WT female mice were treated with selective β3 agonist, CL 316,243, for 2 weeks. Assessments were made for differences in **(A)** NC, UCP1-positive stained perigonadal adipose tissue (PGAT) histology, **(B)** HFD, UCP1-positive stained PGAT, **(C)** UCP1 stain per adipocyte, **(D)** adipocyte size (μm), **(E)** UCP1 protein expression, **(F)** UCP1 mRNA expression. WT, wild-type; KO, ERα knock-out; CTRL, saline control; CL, CL 316,243 treatment; NC, normal chow; HFD, high fat diet; data are expressed as means ± standard error (SE); *n* = 4–10/group. *G* = significant main effect of genotype, *P* < 0.05. *T* = significant main effect of CL, *P* < 0.05, and *D* = significant main effect of diet, *P* < 0.05. G×T = significant interaction between genotype and treatment, *P* < 0.05. G×D = significant interaction between genotype and diet, *P* < 0.05. G×T×D = significant interaction between genotype, treatment, and diet, *P* < 0.05. ^∗^*P* < 0.05 compared to all other groups.

Similar to SQAT, CL-induced beiging was also evident by visual assessment of PGAT UCP1-staining (Figure 6A,B), quantification of UCP1 staining (Figure 6C), reduced adipocyte size (Figure 6D) (T, *P* < 0.001), increased UCP1 protein content (Figure 6E) (T, *P* = 0.005), and increased UCP1 mRNA expression (Figure 6F) in both genotypes. Similar to SQAT, under NC dietary conditions, the lean WT appeared to be more susceptible to CL-induced beiging compared to fatter ERαKO based on the intensity of UCP1+ staining (Figure 6A,B), although this difference did not reach statistical significance, possibly due to small sample size (*n* = 4–5/group). Also similar to SQAT, HFD increased mean adipocyte size (D, *P* < 0.001) and significantly blunted CL-induced beiging in PGAT. Interestingly, ERαKOs had greater “basal” PGAT UCP1 protein (G, *P* = 0.008) than WT, but also experienced greater reductions in UCP1 protein in response to HFD (G×D×T, *P* = 0.011) (Figure 6E). This was likely driven by the heightened CL-induced UCP1 increase in the ERαKO under NC conditions (T×D, *P* = 0.011).

### HFD Increases BAT UCP1 Independently of ERα

**FIGURE 7:**
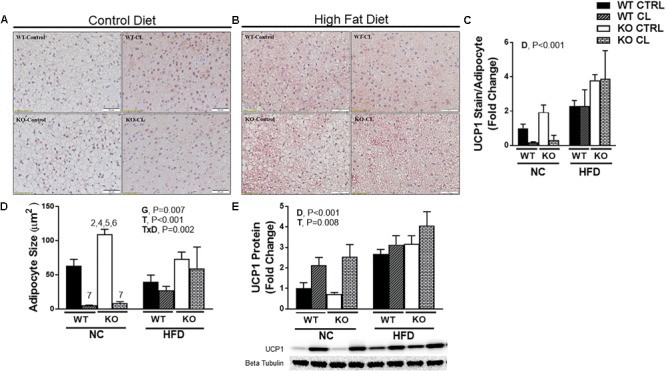
High-fat diet increases UCP1 independently of ERα in BAT. High-fat diet fed ERαKO and WT female mice were treated with selective β3 agonist, CL 316,243, for 2 weeks. Assessments were made for differences in **(A)** NC, UCP1 positive stained interscapular brown adipose tissue (BAT) histology, **(B)** HFD, UCP1-positive stain BAT, **(C)** UCP1 stain per adipocyte, **(D)** adipocyte size (μm), **(E)** UCP1 protein expression. WT, wild-type; KO, ERα knock-out; CTRL, saline control; CL, CL 316,243 treatment; NC, normal chow; HFD, high fat diet; data are expressed as means ± standard error (SE); *n* = 4–10/group. *G* = significant main effect of genotype, *P* < 0.05. *T* = significant main effect of CL, *P* < 0.05. *D* = significant main effect of diet, *P* < 0.05. G×T = significant interaction between genotype and treatment, *P* < 0.05. G×D = significant interaction between genotype and diet, *P* < 0.05. G×T×D = significant interaction between genotype, treatment, and diet, *P* < 0.05.

BAT UCP1+ staining (Figure 7A–C) revealed that the effects of CL were less robust in BAT compared to WAT, confirming what others have reported (Park J.W. et al., 2015). CL significantly reduced BAT mean adipocyte size (Figure 7D) and increased UCP1 levels (Figure 7E) (T, *P* = 0.008). The effects were similar between WT and KO mice. ERαKOs had greater BAT mean adipocyte size than WTs (G, *P* = 0.007) (Figure 7D) possibly suggestive of a “whiter” BAT phenotype. As was the case with the WAT depots, the effects of CL were less robust under HFD conditions (T×D, *P* = 0.002) (Figure 7D). And, as previously reported by our group and others (Feldmann et al., 2009; Cannon and Nedergaard, 2010; Yao et al., 2014; Sakamoto et al., 2016; Winn et al., 2017), HFD significantly increased UCP1 protein (D, *P* < 0.001) (Figure 7E). Here, we illustrate for the first time that this HFD-induced increase in BAT UCP1 content occurs independently of ERα (T, *P* < 0.001).

### Effects of CL and HFD on Blood Biochemistry

Adiponectin is an insulin-sensitizing adipokine (Berg et al., 2001; Fruebis et al., 2001; Yamauchi et al., 2001), and changes in adiponectin production and/or sensitivity has been proposed as a potential mechanism behind CL’s insulin sensitizing effects. Others have observed increases in adiponectin (Fu et al., 2007) and adiponectin receptors (Fu et al., 2008) following CL administration. In our study, we observed CL-induced increases in circulating adiponectin only under HFD conditions (T×D, *P* = 0.037) (Table 2). In fact, whereas ERαKOs had lower circulating adiponectin than WTs (G, *P* = 0.013), that genotype difference was only present under NC diet conditions (G×D, *P* = 0.008). CL did not affect fasting levels of insulin or glucose but reduced fasting NEFAs in both genotypes and dietary conditions (T, *P* = 0.004) (Table 2).

### CL Increases ERβ Expression in WAT

**FIGURE 8:**
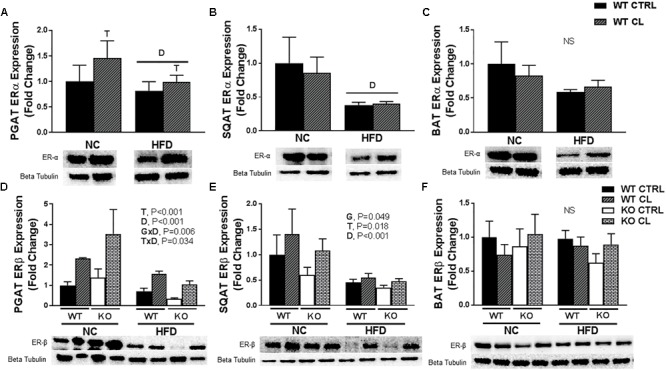
CL 316,243 increases ERβ expression in WAT depots. High-fat diet fed ERαKO and WT female mice were treated with selective β3 agonist, CL 316,243, for 2 weeks. Assessments were made for differences in **(A)** perigonadal adipose tissue (PGAT) ERα protein expression, **(B)** subcutaneous adipose tissue ERα protein expression, **(C)** interscapular brown adipose tissue (BAT) ERα protein expression, **(D)** PGAT ERβ protein expression, **(E)** SQAT ERβ protein expression, **(F)** BAT ERβ protein expression. WT, wild-type; KO, ERα knock-out; CTRL, saline control; CL, CL 316,243 treatment; NC, normal chow; HFD, high fat diet; data are expressed as means ± standard error (SE); *n* = 4–10/group. *G* = significant main effect of genotype, *P* < 0.05. *T* = significant main effect of CL, *P* < 0.05. *D* = significant main effect of diet, *P* < 0.05. G×T = significant interaction between genotype and treatment, P < 0.05. G×D = significant interaction between genotype and diet, *P* < 0.05. G×T×D = significant interaction between genotype, treatment, and diet *P* < 0.05.

To test the hypothesis that CL may improve adipocyte health by suppressing inflammation, we measured several inflammatory markers in various adipose tissue depots, but found no evidence of an anti-inflammatory effect of CL (SQAT inflammatory genes shown in Supplementary Figure S1). Next, we assessed how HFD and CL might affect ERα levels in the adipose tissues of WT mice (Figure 8). We confirmed previous findings (Gorres et al., 2011) that HFD significantly reduces ERα levels in WAT (Figure 8A,B). This was specific to WAT, as HFD did not affect BAT ERα levels (Figure 8C). Interestingly, CL treatment increased PGAT ERα levels and also increased in ERβ protein expression in both WAT depots (PGAT – T, *P* < 0.001 and SQAT – T, *P* = 0.018; Figure 8D,E) but not in BAT (Figure 8F). In line with what happened with UCP1 in PGAT, HFD mitigated the response of CL to increase ERβ (T×D, *P* = 0.034) and there was also a main effect of HFD to decrease ERβ expression in both WAT depots (both D, *P* < 0.001), an effect that also occurred in both genotypes. Notably, ERαKOs were more susceptible to HFD-mediated reduction in PGAT ERβ expression compared to WTs (G×D, *P* = 0.006) which coincided with their overall higher susceptibility to HFD-induced metabolic dysfunction.

## Discussion

β3 adrenergic activation via the compound, CL 314,243 (i.e., CL) restored metabolic health in a rodent model of menopause-associated metabolic dysfunction, the whole-body ERαKO mouse. CL caused WAT beiging, indicated by a CL-induced increase in UCP1, and this correlated with systemic metabolic improvements such as reduced adiposity and improved insulin sensitivity, indicating that the ability of CL to induce adipocyte beiging and improve systemic metabolism does not require ERα. Another notable novel finding was that CL increased WAT ERβ expression, and this occurred similarly in both ERαKO and WT mice. Although the relationship between CL treatment and ERβ expression observed herein is only correlative, the possibility exists that this steroid receptor may play a role in mediating CL’s beneficial effects. Future studies should further interrogate the mechanism driving the increase in ERβ as well as the potential role that ERβ plays in CL-mediated benefits. Taken together, our findings demonstrate that a short 2-week daily treatment with CL effectively restores metabolic health in a model of menopause-associated metabolic dysfunction, the ERαKO mouse.

Previous studies have shown that ERα ablation causes obesity and insulin resistance (Heine et al., 2000; Ohlsson et al., 2000; Davis et al., 2013). Furthermore, activation of ERα signaling protects female mice from diet-induced obesity (Yasrebi et al., 2016). And, studies investigating the role of brain ER signaling have demonstrated that central ER signaling protects against energy balance disturbances. Importantly, our previous studies show that weight gain following ovarian hormone loss is driven specifically by reduced energy expenditure. Here we confirm those findings, demonstrating while ERαKO do not consume more total energy when fed normal rodent chow (NC), they still experience significant weight gain due to suppressed energy expenditure. In addition, we found that the ERαKO overconsume energy when fed HFD and exemplify enhanced energy efficiency on HFD compared to WT, suggesting that the increased weight gain in ERαKO upon HFD feeding is twofold, due to increased energy intake and enhanced energy efficiency. Thus, we confirmed that female ERαKO mice are metabolically impaired compared to their WT counterparts under typically “healthy” NC feeding conditions (Ohlsson et al., 2000; Ribas et al., 2010; Gao and Dahlman-Wright, 2013), and this is exacerbated under HFD feeding, validating that ERα is protective against metabolic dysfunction. This is critical since levels of this steroid receptor diminish following menopause in humans, and this may be responsible for the significant increase in metabolic dysfunction, increasing risk for diabetes and cardiovascular disease among aging women.

ERαKO animals exhibited improvements in body weight, adiposity, and insulin sensitivity following CL, normalizing them to the WT/CTRL. In order to determine if the metabolic improvements attributed to CL treatment differ between WT and ERαKO mice, we fed WT (and ERαKO) HFD in order to induce weight gain and metabolic dysfunction in both genotypes. Confirming many other reports (Ogawa et al., 2003; Li and Krashes, 2015; Park Y.M. et al., 2015), we observed reductions in SPA (G, *P* < 0.001) (Figure 4C) and TEE (G, *P* = 0.023) (Figure 4D) in the ERαKO animals in the rodent active period. Since energy intake was not affected, this reduction in SPA resulted in weight gain in those ERαKO mice (G, *P* < 0.001). However, CL-induced increase in TEE (T, *P* < 0.05) (Figure 4D) due to increased REE (T, *P* = 0.026) (Figure 4E) rescued that increase in body weight and reduced adiposity in the ERαKO.

Under HFD feeding conditions, both WT and ERαKO mice responded positively to CL treatment in terms of attenuation of weight gain and increase in insulin-stimulated glucose clearance. There was a genotype difference in dietary energy overconsumption on HFD, which contributed to the ERαKO gaining more weight on HFD, but CL normalized this in the ERαKO (Figure 4A). This may have contributed to the greater CL-induced weight loss in the ERαKO. Remarkably, CL’s ability to both increase resting energy expenditure (Figure 4E) and reduce HFD energy intake were sufficient to prevent weight gain despite no changes in physical activity (Figure 4C).

Similar to other studies performed in other models (Ghorbani et al., 2012; Poher et al., 2015), we found evidence that CL improved insulin sensitivity, although only surrogate measures of insulin sensitivity were used. A notable experimental problem was that CL induced hypoglycemia during the insulin tolerance testing procedure. While we consider this anecdotal evidence of the insulin-sensitizing effect of CL (none of the vehicle-treated animals experienced hypoglycemia), future studies should perform the gold standard glucose clamp procedure in order to more accurately assess insulin sensitivity. Importantly, the insulin-mediated hypoglycemia occurred in both WT and KO mice (WT *n* = 8; KO *n* = 5), suggesting that both genotypes were sensitive to CL’s effect. While the increase in energy expenditure was likely caused by activation of UCP1 (i.e., adipocyte beiging), the full mechanism responsible for CL-induced improvements remains elusive. In support of the hypothesis that CL-induced beiging of WAT was likely at least partially responsible, we demonstrated CL-induced WAT UCP1 increases in both WT and ERαKO mice. We also observed in both genotypes that animals fed HFD had an attenuated response to CL-induced increases in UCP1 in WAT (Figure 5, 6). Thus, HFD may lessen the therapeutic effectiveness of β3 adrenergic agonists. It is possible that the CL-induced increase in UCP1 may contribute to the insulin-sensitizing effects of CL, although studies need to be conducted in UCP1-null animals in order to directly test this hypothesis. Importantly, we previously demonstrated in female mice that UCP1 has insulin-sensitizing effects that may be independent of adiposity changes (Winn et al., 2017) and respond more adversely to ovariectomy compared to WT controls (Clookey et al., 2018).

Some evidence suggests that ERα may facilitate WAT browning by increasing sensitivity to browning stimuli in progenitor cells (Lapid et al., 2014). This topic was reviewed recently (Frank et al., 2017). Here, we demonstrate that CL improves metabolic health in ERαKO mice, demonstrating that the metabolic benefits of CL do not require ERα. Further, CL-induced browning was indicated by both increased UCP1 content and adipocyte phenotypic changes in both genotypes. However, under NC dietary conditions, visual examination of the WT WAT following CL indicated smaller, more multilocular adipocytes compared to that of the ERαKO/CL suggesting that loss of ERα signaling may impair beiging. Because this genotype difference did not hold true under HFD feeding conditions, the differential response under NC conditions may have been attributed to the greater adiposity in ERαKOs, and not necessarily their lack of ERa. In fact, ERαKOs appeared more sensitive to CL when assessed for total UCP1 protein and RNA expression in response to CL compared to WT (Figure 5, 6). And, responses to CL in the HFD-fed WT and the NC-fed ERαKO (i.e., adjusting for adiposity difference between KO and WT) were strikingly similar.

While cold is the main stimulus for UCP1 increases in BAT, diet-induced obesity also increases BAT UCP1 (Feldmann et al., 2009; Cannon and Nedergaard, 2010; Yao et al., 2014; Sakamoto et al., 2016; Winn et al., 2017). Obesity-induced increases in BAT UCP1 may serve as a means to restore energy balance during energy surplus. We previously observed that ovariectomy-induced obesity also increases BAT UCP1 (Vieira-Potter et al., 2015). However, that finding was surprising to us since other studies have shown that estrogen increases UCP1 gene expression and BAT activity in rodents (Pedersen et al., 2001; Martinez de Morentin et al., 2014) and humans (Valle et al., 2008; Velickovic et al., 2014). Clearly, the relationship between estrogen and UCP1 is complex and far from completely understood. Here, we show that obesity induced by ERα ablation does not significantly increase BAT UCP1, yet HFD-induced obesity did induce BAT UCP1 both in WT and ERαKO mice. CL did not further increase BAT UCP1 under HFD conditions (Figure 7E), perhaps due to a ceiling effect of BAT UCP1. Previous studies have also reported that CL more potently induces UCP1 in WAT (i.e., induces beiging) than BAT and supports the hypothesis that the beneficial effects of CL are mediated more through WAT than BAT (Poher et al., 2015). This is of particular relevance and importance since we have far greater relative abundance of WAT than BAT.

A novel observation made in this present study was that ERβ protein expression also increased with CL, specifically in WAT depots, paralleling what was observed with UCP1. Again, strikingly similar to the effects on UCP1, the ability of CL to increase ERβ was attenuated under conditions of HFD (Figure 8). Notably, ERβ ligands have been shown to reduce body weight and fat mass (Ponnusamy et al., 2016; Gonzalez-Granillo et al., 2019) and rescue ovariectomy-induced obesity (Yepuru et al., 2010). Moreover, ERβ ligands increase oxygen consumption and mitochondrial activity (Ponnusamy et al., 2016), and increase UCP1 protein in BAT (Yepuru et al., 2010; Ponnusamy et al., 2016 #124). Although we did not observe any changes in ERβ in BAT, we did observe increases in this protein in WAT, the depot where CL most strongly induces UCP1 expression. Thus, CL’s effects may be mediated via interaction with ERβ. In thinking about potential mechanisms, this nuclear hormone receptor shares a similar cofactor pool to that of PPARγ, another nuclear hormone receptor (Foryst-Ludwig et al., 2008), which is known to enhance insulin sensitivity and induce adipocyte proliferation. In cell culture, ERβ inhibits PPARγ activity (i.e., adipogenic gene expression and adipogenesis) (Chu et al., 2014) while CL also has been shown to suppress PPARγ (Li et al., 2017), a finding that may explain CL’s ability to suppress adiponectin production, which requires PPARγ. In support of that hypothesis, ERαKO (but not WT) were resistant to HFD-induced decrease in circulating adiponectin levels. It is noteworthy that adiponectin, besides increasing insulin sensitivity, activates central AMPK, which is known to activate feeding centers in the brain and suppress BAT UCP1 levels, thereby enhancing energy storage via hyperphagia and increased energy efficiency (i.e., via reduced diet-induced thermogenesis) (Martinez de Morentin et al., 2014). Thus, in WT mice, HFD-induced reduction in adiponectin levels was consistent with their protection from excess HFD-induced energy intake and HFD-induced BAT UCP1 increase, both observed in the ERαKO. The effect of CL on ERβ and its crosstalk with PPARγ certainly requires further investigation.

The findings of this study should be considered in light of its potential limitations. First, we used the ERαKO model, which still produces ovarian estrogen yet does not have ERα- mediated estrogen signaling in any tissues. This is different from human menopause where women display reduced production of ovarian estrogen and thus reduced signaling, but still have functional ERs. Future studies should compare the efficacy of CL treatment in ovary-intact vs. ovariectomized rodents. Secondly, we chose to conduct our studies at thermoneutrality because cooler temperatures may affect metabolic parameters in rodents. That is, we wanted to isolate the effects of CL from those induced by cold. A recent study showed that C57BL/6J mice are not more susceptible to HFD-induced insulin resistance, but cooler temperatures do cause the rodents to increase energy expenditure (which is coupled with increased intake) (Small et al., 2018). Notwithstanding, future studies should test how environmental temperature affects responses to CL in this animal model. In addition, the studies performed herein were only done in whole body ERαKO and WT mice. Since ERα is expressed in many tissues, and has various developmental roles, it is also critical to perform these studies in conditional knock-out models, where ER can be experimentally suppressed after development, as well as in adipose tissue-specific ER knock out models. Finally, it is important to note that the diets used in this study were not free of soy phytoestrogens, which are weak ER ligands. Thus, interactions between soy phytoestrogens and CL treatment could not be determined in the current study; future studies should determine the role(s) played by endogenous, exogenous, and dietary estrogens.

## Overall Conclusion

Postmenopausal women are at heightened risk for obesity and its related metabolic disorders (Carr, 2003; Dubnov-Raz et al., 2007; Teede et al., 2010), whereas estrogen-sufficient females have superior metabolic health, more relative BAT, and may be more responsive to WAT beiging (Himms-Hagen, 1989; Cypess et al., 2009; Pfannenberg et al., 2010; Ouellet et al., 2011; Kim et al., 2016). Mechanisms are not fully understood, but suppressed estrogen signaling through ERα is thought to play a major role in the adipose tissue dysfunction that follows hormone loss (Heine et al., 2000; Ohlsson et al., 2000; Geary et al., 2001; Liang et al., 2002; Ribas et al., 2010; Davis et al., 2013). We sought to determine if the adipose tissue and systemic metabolic dysfunction caused by loss of estrogen signaling through ERα could be rescued by systemic b_3_ adrenergic receptor activation via CL 316,243, a known b_3_ adrenergic receptor agonist. Further, we determined if the effectiveness of this drug to induce WAT beiging and improve metabolism varies in the presence and absence of systemic ERα and under normal chow and high-fat (i.e., Western style) dietary conditions. We discovered that CL effectively rescues HFD- and ERα ablation-induced metabolic dysfunction, and that ERα-null animals may be more sensitive to WAT UCP1 induction. Finally, HFD feeding may interfere with CL’s effectiveness to activate UCP1, which may be an important consideration for utilizing β_3_ adrenergic agonists as a therapeutic for obesity.

## Author Contributions

SC, RW, DS, and MW performed the experiments. SC, RW, and VV-P analyzed the data. SC and VV-P interpreted the results and prepared the manuscript. SC and RW prepared the figures. SC, RW, JP, DL, KF, RR, and VV-P revised and edited the manuscript. SC, RW, DS, MW, DL, JP, KF, RR, and VV-P approved final version of the manuscript. VV-P conceived and designed the research.

## Conflict of Interest Statement

The authors declare that the research was conducted in the absence of any commercial or financial relationships that could be construed as a potential conflict of interest.

## Supplementary Material

FIGURE S1 Normal chow and high-fat diet comparison of body weight, energy intake, depot weights, adipose tissue inflammatory gene expression, adipose tissue UCP2 protein content, liver triglycerides, and insulin resistance. Normal chow and high-fat diet fed ERαKO and WT female mice were treated with selective β3 agonist, CL 316,243, for 2 weeks. Assessments were made for differences in **(A)** body weight, **(B)** energy intake, **(C)** subcutaneous adipose tissue (SQAT) depot weight, **(D)** perigonadal adipose tissue (PGAT) depot weight, **(E)** brown adipose tissue (BAT) depot weight, **(F)** HOMA-IR, **(G)** ADIPO-IR, **(H)** subcutaneous adipose tissue integrin (CD11c) gene expression, **(I)** subcutaneous adipose tissue tumor necrosis factor alpha (TNFα) gene expression, **(J)** perigonadal adipose tissue (PGAT) UCP2 expression, **(K)** subcutaneous adipose tissue (SQAT) UCP2 expression, **(L)** brown adipose tissue (BAT) UCP2 expression. **(M)** liver triglyceride content. WT, wild-type; KO, ERα knock-out; CTRL, saline control; CL, CL 316,243 treatment; NC, normal chow; HFD, high fat diet; data are expressed as means ± standard error (SE); *n* = 4–10/group. G, significant main effect of genotype, *P* < 0.05. T, significant main effect of CL, *P* < 0.05. D, significant main effect of diet, *P* < 0.05. GxT, significant interaction between genotype and treatment, *P* < 0.05. GxD, significant interaction between genotype and diet, *P* < 0.05. GxTxD, significant interaction between genotype, treatment, and diet, *P* < 0.05.
