# Supplementary figures and images for: Beta 3 Adrenergic Receptor Activation Rescues Metabolic Dysfunction in Female Estrogen Receptor Alpha-Null Mice
Source: Front Physiol. 2019 Feb 5;10:9. doi: 10.3389/fphys.2019.00009 (PMC6371032; doi:10.3389/fphys.2019.00009)

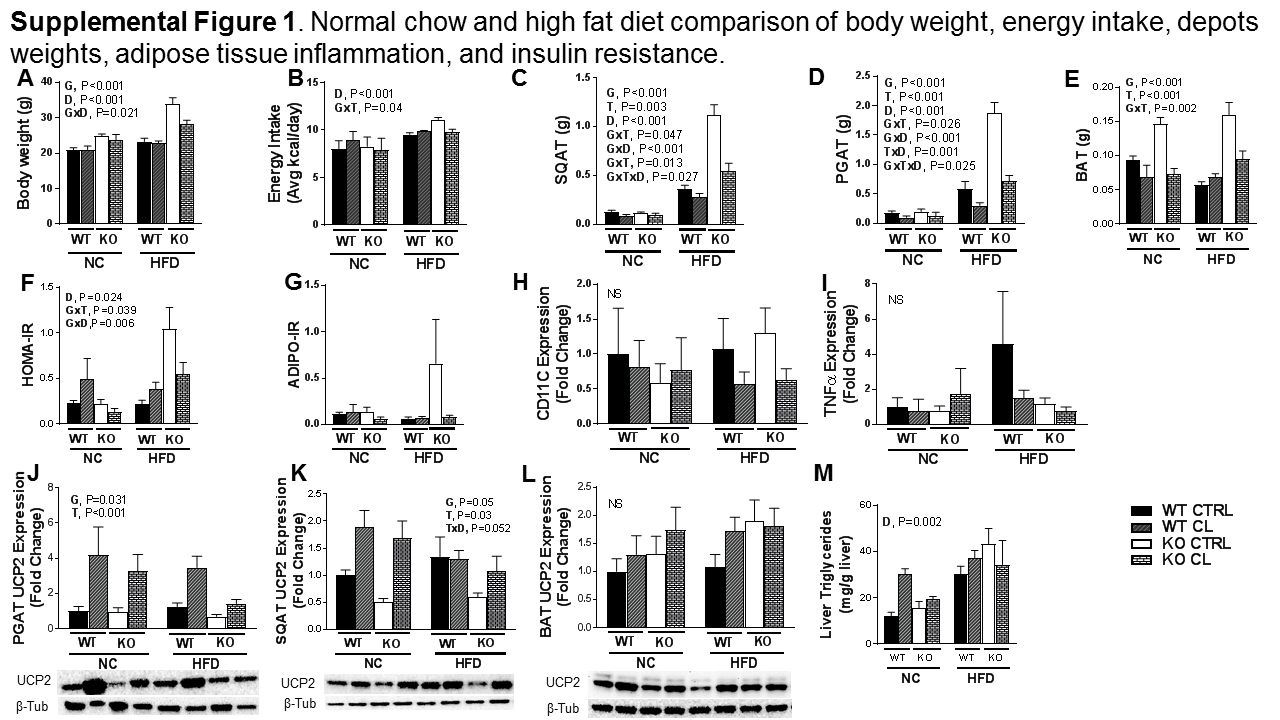

Supplement: FIGURE S1 — Normal chow and high-fat diet comparison of body weight, energy intake, depot weights, adipose tissue inflammatory gene expression, adipose tissue UCP2 protein content, liver triglycerides, and insulin resistance. Normal chow and high-fat diet fed ERαKO and WT female mice were treated with selective β3 agonist, CL 316,243, for 2 weeks. Assessments were made for differences in (A) body weight, (B) energy intake, (C) subcutaneous adipose tissue (SQAT) depot weight, (D) perigonadal adipose tissue (PGAT) depot weight, (E) brown adipose tissue (BAT) depot weight, (F) HOMA-IR, (G) ADIPO-IR, (H) subcutaneous adipose tissue integrin (CD11c) gene expression, (I) subcutaneous adipose tissue tumor necrosis factor alpha (TNFα) gene expression, (J) perigonadal adipose tissue (PGAT) UCP2 expression, (K) subcutaneous adipose tissue (SQAT) UCP2 expression, (L) brown adipose tissue (BAT) UCP2 expression. (M) liver triglyceride content. WT, wild-type; KO, ERα knock-out; CTRL, saline control; CL, CL 316,243 treatment; NC, normal chow; HFD, high fat diet; data are expressed as means ± standard error (SE); n = 4–10/group. G, significant main effect of genotype, P < 0.05. T, significant main effect of CL, P < 0.05. D, significant main effect of diet, P < 0.05. GxT, significant interaction between genotype and treatment, P < 0.05. GxD, significant interaction between genotype and diet, P < 0.05. GxTxD, significant interaction between genotype, treatment, and diet, P < 0.05. [file Image_1.TIF]
